# P-2361. Real-World Experience of Letermovir for CMV Prophylaxis in Solid Organ Transplant Recipients

**DOI:** 10.1093/ofid/ofae631.2512

**Published:** 2025-01-29

**Authors:** Emily Perez, Alexandra Cheezem, Pierina Cabrera-Rios, Molly Macek, Ennie Cano, Lilian M Abbo, Christine A Vu

**Affiliations:** Jackson Health System, Miami, Florida; Jackson Health System, Miami, Florida; Jackson Health System, Miami, Florida; Jackson Health System, Miami, Florida; Jackson Health System, Miami, Florida; University of Miami Miller School of Medicine, Jackson Health System, Aventura, FL; Jackson Memorial Hospital, Miami, Florida

## Abstract

**Background:**

Valganciclovir is recommended as first-line for cytomegalovirus (CMV) prophylaxis after solid organ transplant, however, its use may be limited by myelosuppression. In 2023, letermovir was approved for CMV prophylaxis in high-risk kidney transplant recipients. The aim of this study was to describe our real-world experience utilizing letermovir for CMV prophylaxis in select solid organ transplant recipients.

**Figure:**
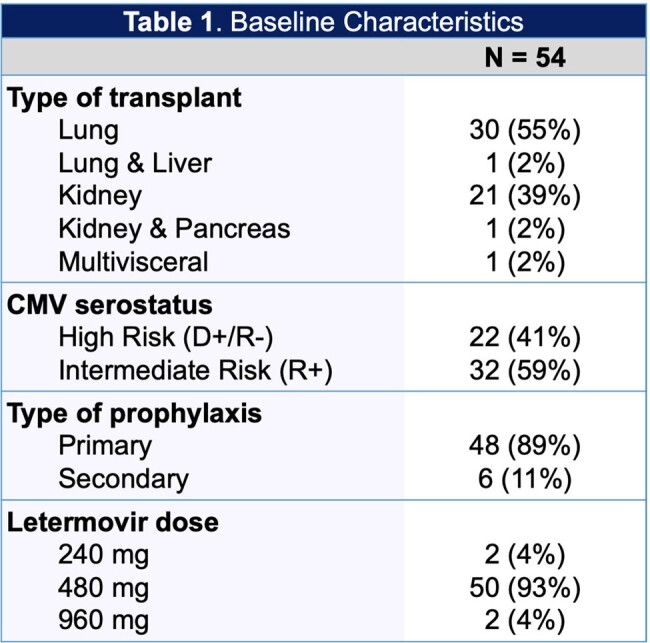

**Methods:**

A single-center, retrospective study was conducted in adult solid organ transplant recipients who received letermovir for CMV prophylaxis between December 2022 and March 2024 at the Miami Transplant Institute. We assessed white blood cell count (WBC) recovery following the switch from valganciclovir to letermovir, growth-colony stimulating factor (G-CSF) use, drug interactions, CMV DNAemia, and outpatient accessibility. Data was analyzed using descriptive statistics.

**Figure:**
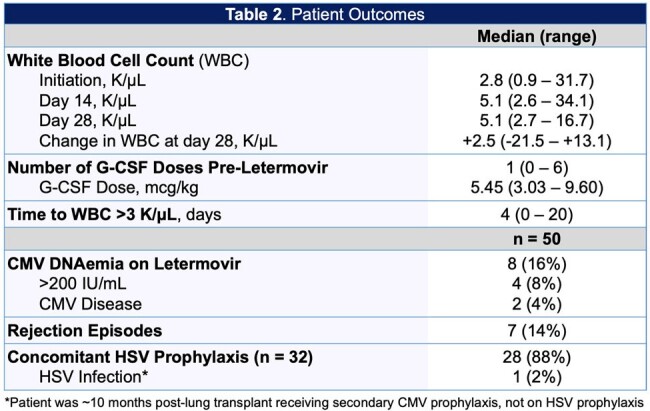

**Results:**

Of the 54 patients included, 31 (57%) received a lung transplant, 22 (41%) received a kidney transplant, and 1 (2%) received a multivisceral transplant. For patients switched from valganciclovir to letermovir, the median WBC change at day 28 was +2.5 k/uL, 95% of patients achieved WBC recovery. Median number of G-CSF doses prior to letermovir was 1 with an average dose of 5.65 mcg/kg. For patients receiving tacrolimus/cyclosporine, 27 (54%) required a dose reduction after starting letermovir. We had 2 cases of breakthrough CMV disease. Of the patients requiring prophylaxis for herpes simplex virus (HSV), 4 (13%) patients were not prescribed acyclovir/valacyclovir. For outpatient accessibility, 27 (59%) of patients required prior authorizations, four (9%) patients were unable to start letermovir due to lack of insurance approval, and 1 (2%) patient discontinued treatment due to ongoing insurance issues.

**Figure:**
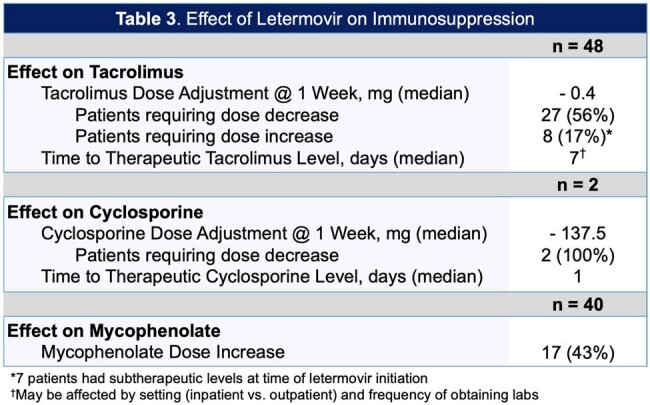

**Conclusion:**

This study supports the use of letermovir to prevent CMV infection in high- and intermediate-risk transplant recipients. Most patients who were switched from valganciclovir experienced WBC recovery. Our more notable findings were the challenges faced with prescribing letermovir. Early pharmacy engagement may be necessary to help address barriers related to cost, insurance coverage, drug-drug interactions, and reinforce concomitant prescribing of HSV prophylaxis.

**Figure:**
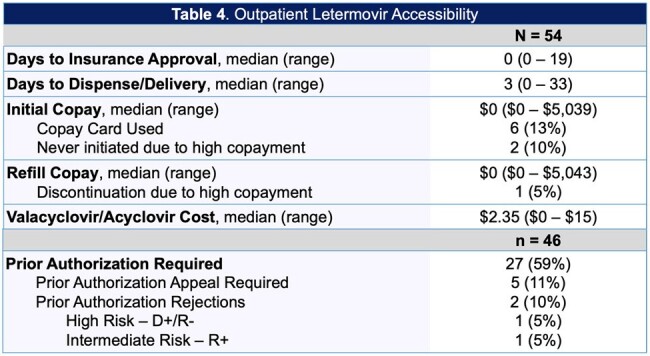

**Disclosures:**

All Authors: No reported disclosures

